# An analytic solution of full-sky spherical geometry for satellite relative motions

**DOI:** 10.1038/s41598-021-88483-2

**Published:** 2021-04-27

**Authors:** Soung Sub Lee, Christopher D. Hall

**Affiliations:** 1 Department of Aerospace System Engineering, Sejong University, Seoul, 05006 South Korea; 2 Mechanical Engineering Department, The University of New Mexico, Albuquerque, NM 87131 USA

**Keywords:** Astronomy and astrophysics, Aerospace engineering

## Abstract

Herein, an exact and efficient analytic solution for an unperturbed satellite relative motion was developed using a direct geometrical approach. The derivation of the relative motion geometrically interpreted the projected Keplerian orbits of the satellites on a sphere (Earth and celestial spheres) using the solutions of full-sky spherical triangles. The results were basic and computationally faster than the vector and plane geometry solutions owing to the advantages of the full-sky spherical geometry. Accordingly, the validity of the proposed solution was evaluated by comparing it with other analytic relative motion theories in terms of modeling accuracy and efficiency. The modeling accuracy showed an equivalent performance with Vadali’s nonlinear unit sphere approach, which is essentially equal to the Yan–Alfriend nonlinear theory. Moreover, the efficiency was demonstrated by the lowest computational cost compared with other models. In conclusion, the proposed modeling approach illustrates a compact and efficient closed-form solution for satellite relative motion dynamics.

## Introduction

The dynamic models of the satellite relative motion are diverse and the developed solutions have paid attention to the performance of the dynamic model, such as computational complexity, accuracy, types of perturbations, and model assumptions. The main focus of these dynamic models has been on the study of formation flying and rendezvous and docking maneuvers of satellites. For these applications, the satellite relative motion theories began with the equations of motion derived by Clohessy and Wiltshire^1^. The reference satellite orbit was assumed to be circular, and the relative orbit coordinates were small compared with the reference orbit radius; hence, the resulting equation of motion was linearized. Lawden^2^ found an improved form for the relative motion, including reference orbit eccentricity. Carter^3^ later extended Lawden’s solution. Subsequently, Kechichian^4^ developed the exact formulation for a general elliptic orbit to analyze the relative motion in the presence of *J*_2_ potential and atmospheric drag. However, the resulting equations were required to use numerical integration over time. Sedwick et al.^5,6^ applied the *J*_2_ potential forcing function to the right-hand side of Hill’s equations. Schweighart followed these equations and found analytic solutions. Melton^7^ later developed an approximate solution, thereby expanding the state transition matrix in powers of eccentricity with a time-explicit representation.

Accordingly, numerous theories of satellite relative motion have been added to the literature in the last decades. A brief survey was published by Alfriend and Yan^8^, who compared and evaluated various relative motion theories: Hill’s equations, the Gim–Alfriend state transition matrix^9^, the small-eccentricity state transition matrix^8^, the non-*J*_2_ state transition matrix^8^, the unit sphere approach (USA)^10^, and the Alfriend–Yan nonlinear method^11^. Their evaluation of the results showed that the USA and the Yan–Alfriend nonlinear method present the highest accuracy for all eccentricities and relative orbit sizes. The USA was proposed by Vadali, who achieved the exact analytic expression in terms of differential orbital elements for relative motion problems. Alfriend and Yan applied the geometrical method to the nonlinear relative motion. The method was employed in long-term prediction of mean orbital elements, including nonlinear *J*_2_ effects, and in transforming Hill’s frame. Most of the abovementioned relative motion theories have been developed using the formation flying concept for station keeping.

Recently, a comprehensive survey and assessment of satellite relative motion dynamics models was conducted by Joshua Sullivan^12^. This comparative study provided an extensive overview of the currently available literature and assessed the newly developed relative motion solutions, adding to the comparisons of Alfriend and Yan: the curvilinear Hill–Clohessy–Wiltshire (HCW) model^13^, the quadratic Volterra model^14^, GAMSTM^15^, KGDSTM^16^, and the Biria–Russell Vinti method^17^. The goal of this paper is to study several key features of the dynamics models paying attention to dynamical state representations, model assumptions, types of perturbations, accuracy, and computational complexity. Finally, several meaningful comparative results are presented.

In previous works, the analyst was forced into analytic approaches using vector terms rather than spherical geometry because of the singularity problem. However, these problems have been eliminated by solutions of full-sky triangles and plugged into computer programming languages for many years. This study aims to develop a solution for relative motion dynamics by direct geometrical interpretation using the advantages of full-sky spherical geometry solutions. Spherical geometry now makes computer implementation quite simple, and this has been discussed in Ref.^18^.

To derive the equations of motion, the approach geometrically interprets the projected base and the target satellite Keplerian orbits on a sphere (Earth and celestial spheres) with the spherical trigonometry solutions. The resulting equations are expressed as azimuth and elevation angles with their derivatives representing the relative angular position and velocity of the target satellite with respect to the base satellite. Subsequently, the azimuth and elevation angles are then transformed into the associated rectangular coordinates for the relative position and velocity vectors in the base satellite centered frame. The proposed solution is validated by modeling accuracy and efficiency compared with those of the exact analytic dynamic models of the satellite relative motion theories.

## Keplerian orbit in spherical coordinate systems

We project a Keplerian orbit on a celestial sphere using spherical coordinates. The Keplerian orbit is commonly specified by the classical orbital elements for state representations in space^19^. The six orbital element sets are as follows:

1 $$\left[a,e,\Omega ,i,\omega ,\nu \right]$$

**Figure 1:**
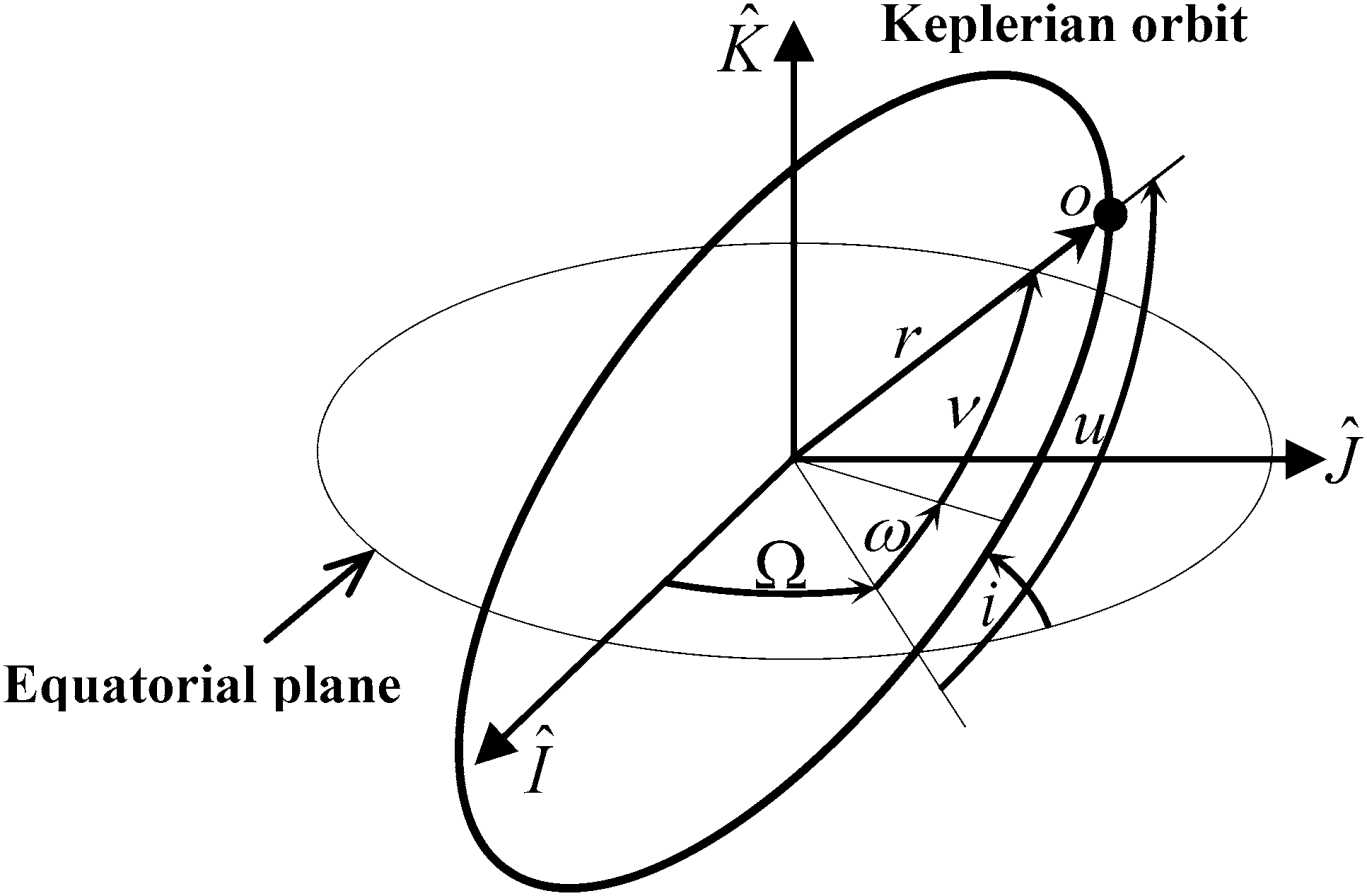
Keplerian orbit elements.

The semi-major axis, *a*, and the eccentricity, *e*, listed as the first two elements above describe the orbit size and shape, respectively, while Ω, *i*, and *ω* define the orbit plane orientation. The final classical orbital element is the true anomaly, *ν*, which determines the object’s current angular position relative to the perigee. Figure 1 illustrates the orbit elements Ω*, i, ω,* and *ν*, which are angle-related orbit elements that describe the Keplerian orbit from the center of the Earth.

**Figure 2:**
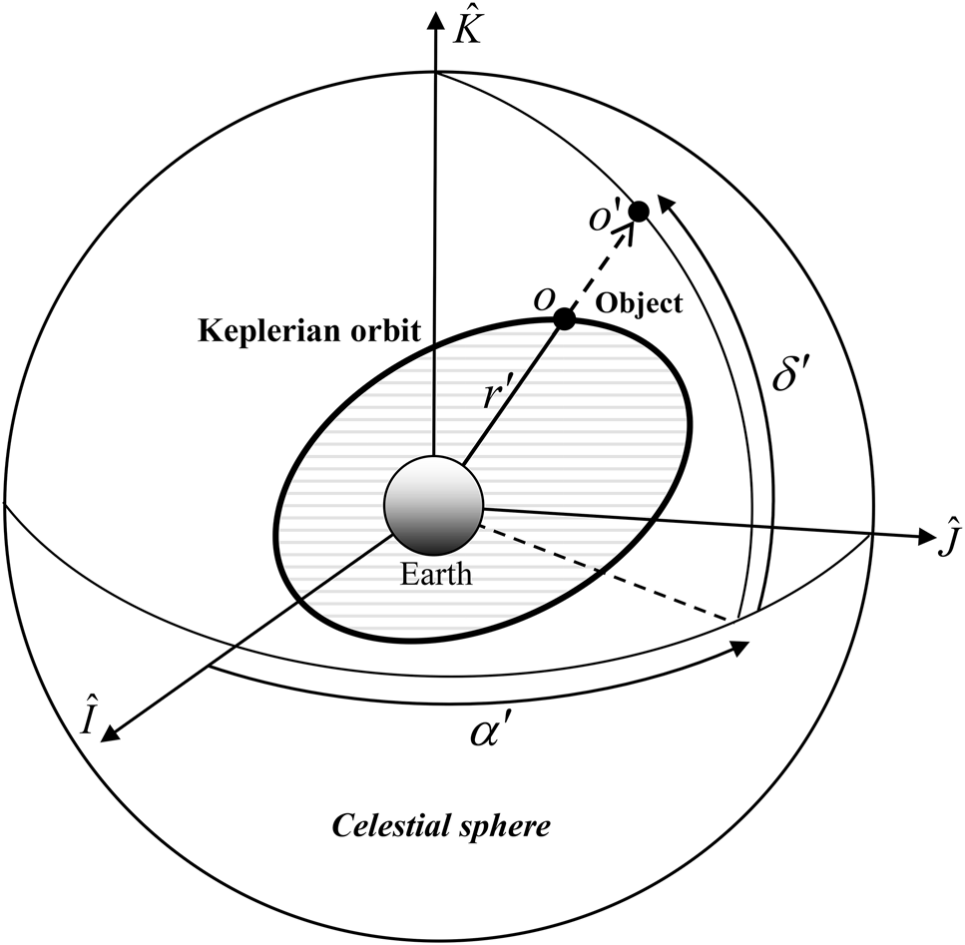
Projection of a Keplerian orbit on a celestial sphere.

A spherical coordinate system in space can be used to represent the Keplerian orbit projected on a sphere. In Fig. 2, $\widehat{I}$ axis is along the vernal equinox direction and the $\widehat{K}$ axis is in the direction of the north pole, and then the elevation angle, ${\delta }^{^{\prime}}$, defines the angle between the straight line from the center of the Earth to ${O}^{^{\prime}}$ and the projection of this line on the $\widehat{I} \widehat{J}$ planeThe angle between this projection and the $\widehat{I}$ axis is defined as the azimuth angle, ${\alpha }^{^{\prime}}$. The formulas for ${\alpha }^{^{\prime}}$ and ${\delta }^{^{\prime}}$ can be expressed as follows in terms of the angle-related orbital elements if we represent the projected Keplerian orbit by ${\alpha }^{^{\prime}}$ and ${\delta }^{^{\prime}}$:

2 $$\begin{array}{l}{\alpha }^{^{\prime}}=f\left(\nu ; i,\Omega , \omega \right)\\ {\delta }^{^{\prime}}=g\left(\nu ; i,\Omega , \omega \right)\end{array}$$

Angles ${\alpha }^{^{\prime}}$ and ${\delta }^{^{\prime}}$ and the radial distance, *r*, of the object determine the spherical coordinate system in space. The radial distance, *r*, is written as follows in terms of *ν*:

3 $$r=\frac{a\left(1-{e}^{2}\right)}{1+e\mathrm{cos}\nu }$$

If we represent the position of the object in the rectangular coordinate system, the spherical coordinate system is transformed by the following relations:

4 $${r}_{\widehat{I} \widehat{J} \widehat{K} }= \left(\begin{array}{l}rcos{\delta }^{^{\prime}}cos{\alpha }^{^{\prime}}\\ rcos{\delta }^{^{\prime}}sin{\alpha }^{^{\prime}}\\ rcos{\delta }^{^{\prime}}\end{array}\right)$$

Angles ${\alpha }^{^{\prime}}$ and ${\delta }^{^{\prime}}$ are expressed in the next section in terms of the angle-related orbit elements using direct geometrical interpretations.

## Geometrical relative orbit modeling

In this section, we geometrically derive the relative position and the velocity vectors of a target satellite relative to the base satellite. The subscript B denotes the base satellite while subscript T denotes the target satellite.

**Figure 3:**
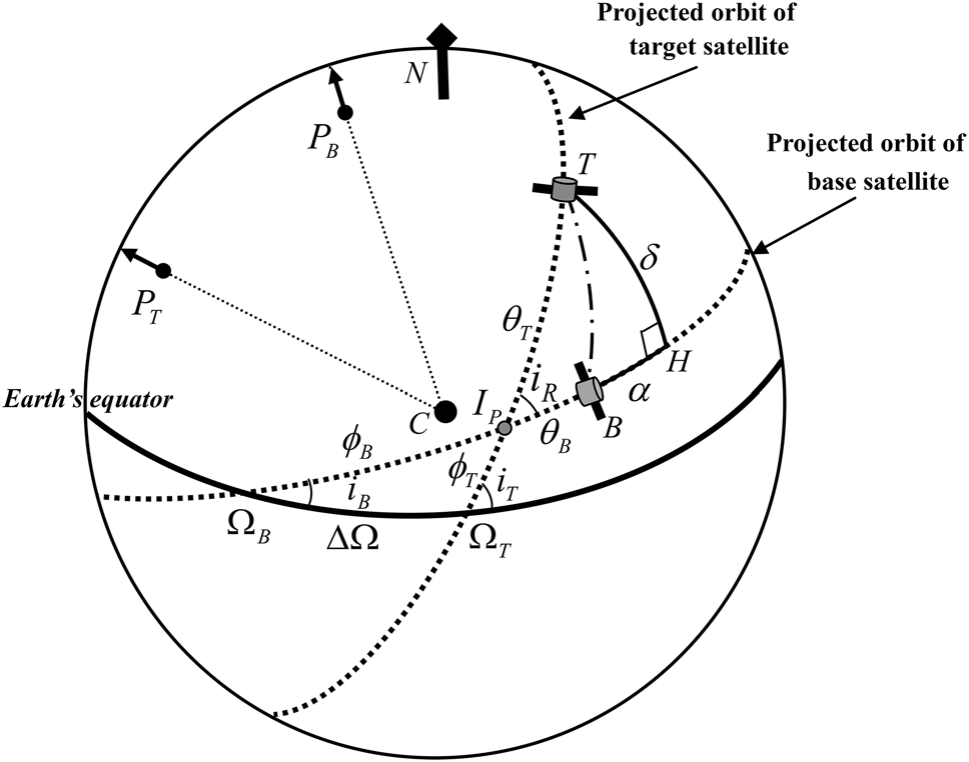
Geometry for modeling the relative motion on the surface of a sphere.

The Keplerian orbits of the two satellites are projected on a sphere for geometrical interpretation, as seen in Fig. 3. ${P}_{B}$ and ${P}_{T}$ denote the orbit poles of the satellites. The dotted lines on the sphere represent the projected Keplerian orbits of the two satellites and the solid line represents an equatorial plane. The intersection point, ${I}_{P}$, is defined as the projected crossing point of the two orbit planes on the surface.

### Note 1

In spherical geometry, intersection points always exist between great circle arcs on the sphere if they do not have the same inclinations.

The relative position of the target satellite, *T*, with respect to the base satellite, *B*, is given by the azimuth angle, *α*, and the elevation angle, *δ*. Angle *α* is perpendicular to angle *δ* through point *H*.

We introduce the argument of latitudes for the transformation between the classical orbital elements and the angular positions on the sphere. The argument of latitudes, ${u}_{B,T}$, measures the arc lengths from the ascending nodes to the current satellite angular position. On the sphere, ${u}_{B,T}$ can be expressed as

5 $${u}_{j}={\phi }_{j}+{\theta }_{j}={\omega }_{j}+{\nu }_{j} j= B, T$$

where the arc lengths ${\phi }_{j}$ and ${\theta }_{j}$ represent the distance from the ascending nodes, ${\Omega }_{j}$, to the intersection point, ${I}_{P}$, and from ${I}_{P}$ to the satellite’s current angular position, respectively. For the derivation of the satellite relative motion, a key parameter is the relative inclination, ${i}_{R}$, which is the angle between two orbit planes at ${I}_{P}$. We use the spherical triangle $\Delta {\Omega }_{B }{\Omega }_{T}{I}_{P}$ to compute ${i}_{R}$. Because ${i}_{R}$ is not equal to the difference between two inclinations of the orbits (i.e., ${i}_{R}\ne {i}_{T}-{i}_{B}$), we must apply the law of cosines for angles to the triangle:

6 $$\mathrm{cos}{i}_{R}=\mathrm{cos}{i}_{B}cos{i}_{T}+\mathrm{sin}{i}_{B}\mathrm{sin}{i}_{T}\mathrm{cos}\Delta \Omega $$

where, the relative ascending node, ∆Ω, is defined as follows:

7 $$\Delta \Omega = {\Omega }_{T} - {\Omega }_{B}$$

**Figure 4:**
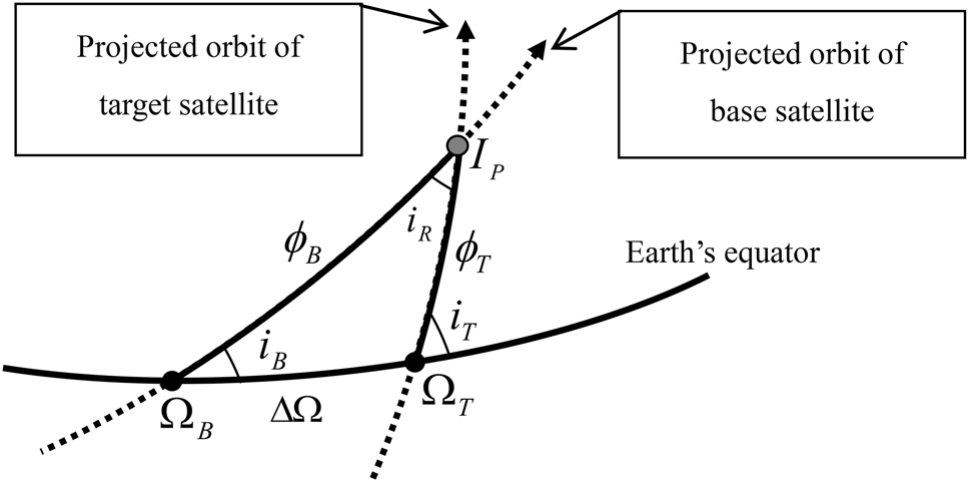
Spherical triangle for computing ${\mathbf{\varnothing }}_{\mathbf{B}}$ and ${\mathbf{\varnothing }}_{\mathbf{T}}$.

We first derive *α* and *δ* for the target satellite relative to the base satellite in terms of the angle-related orbit elements Ω*, i, ω*, and *ν*. The general solutions for spherical triangles are given in Ref.^18^. The spherical triangle $\Delta {\Omega }_{B }{\Omega }_{T}{I}_{P}$ in Fig. 3 is taken to solve ${\phi }_{B}$ and ${\phi }_{T}$. Figure 4 shows a detailed view of the spherical triangle. We apply the law of sines to the spherical triangle to compute $\mathrm{sin }{\phi }_{B}$:

8 $$\mathrm{sin }{\upphi }_{\mathrm{B}}=\frac{\mathrm{sin }\Delta \Omega \mathrm{ sin }{i}_{T}}{\mathrm{sin }{i}_{R}}$$

Applying the law of cosines for angles to the spherical triangle $\Delta {\Omega }_{B }{\Omega }_{T}{I}_{P}$, we find another geometrical relationship to compute $\mathrm{cos }{\phi }_{B}$:

9 $$\mathrm{cos}{ \phi }_{B}=\frac{\mathrm{cos}\left(180-{i}_{T}\right)+\mathrm{cos}{ i}_{B }\mathrm{cos }{i}_{R}}{\mathrm{sin}{ i}_{B}\mathrm{ sin }{i}_{R}}$$

Dividing Eqs. (8) by (9) and applying the full-sky trigonometry solutions give

10 $${\phi }_{B}=\mathrm{atan}2 \left[\mathrm{sin}\Delta \Omega \mathrm{sin}{i}_{B}\mathrm{ sin}{i}_{T}, -\mathrm{cos}{i}_{T}+\mathrm{cos}{i}_{B}\mathrm{ cos}{i}_{R}\right]$$

To compute $\mathrm{sin}{ \phi }_{T}$, the law of sines is also applied to the spherical triangle seen in Fig. 4:

11 $$\mathrm{sin}{ \phi }_{T} = \frac{\mathrm{sin }\Delta \Omega \mathrm{sin }{i}_{B}}{sin {i}_{R}}$$

Using the law of cosine for angles, we also obtain

12 $$\mathrm{cos }{\phi }_{T} = \frac{\mathrm{cos }{i}_{B}+\mathrm{cos}\left(180-{i}_{T}\right) \mathrm{cos }{i}_{R}}{\mathrm{sin}\left(180-{i}_{T}\right) \mathrm{sin }{i}_{R}}$$

Dividing Eqs. (11) by (12) results in

13 $${\phi }_{T} = \mathrm{atan}2 \left[\mathrm{sin }\Delta \Omega \mathrm{sin }{i}_{B }\mathrm{sin }{i}_{T}, \mathrm{cos }{i}_{B}-\mathrm{cos }{i}_{T}\mathrm{cos }{i}_{R}\right]$$

**Figure 5:**
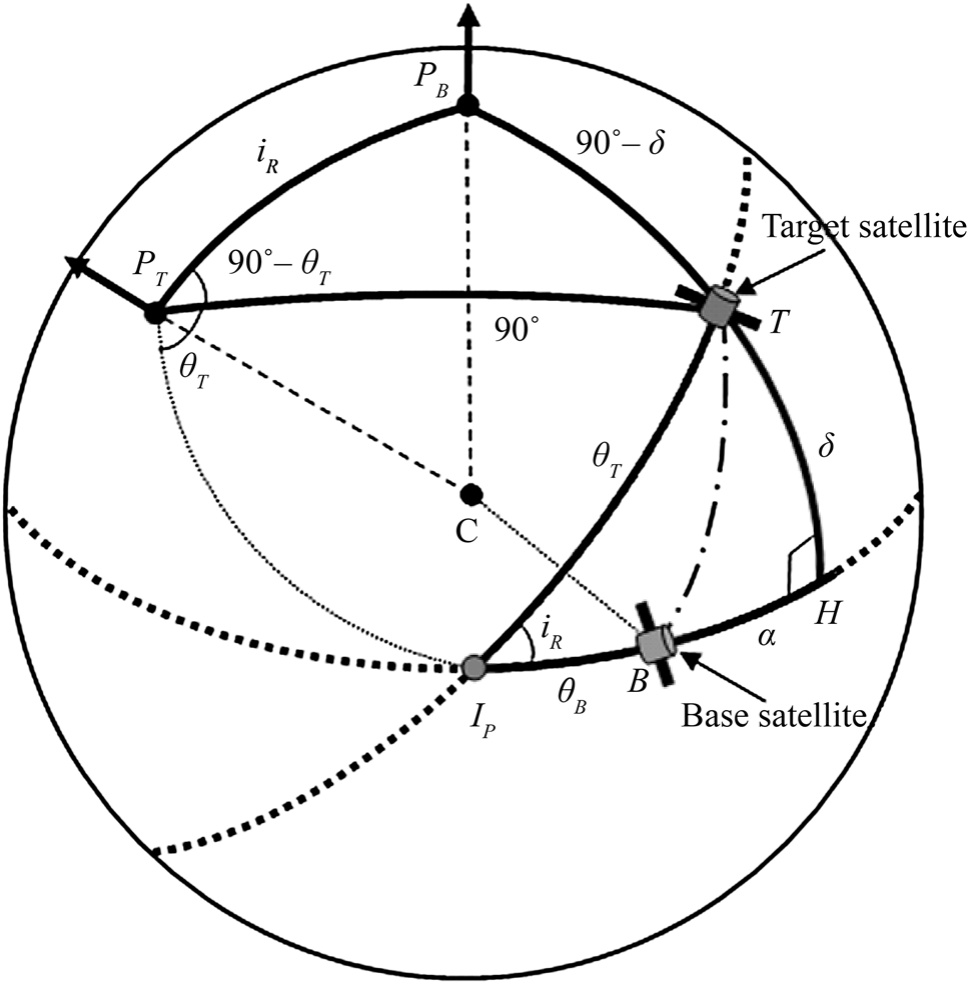
Geometry for computing α and δ with ∆Ω = 0.

Now we consider the spherical triangles on the surface of the sphere with ∆Ω = 0. In this case, we construct a celestial sphere having the pole ${P}_{B}$ of the base satellite as a geographical pole, shown in Fig. 5. The celestial sphere has two spherical triangles, $\Delta {P}_{B}{P}_{T}T$ and $\Delta \mathrm{T}H{I}_{P}$. Note that the angle $\Delta {P}_{B}{P}_{T}{I}_{P}$ is always 90°, regardless of the inclination of the satellites. The angle T ${P}_{B}{I}_{P}$ is equivalent to the angle ${\theta }_{T}$ by applying the law of sines. Hence, the angle ${P}_{B}{P}_{T}T$ is obtained by subtracting ${\theta }_{T}$ from 90°. The arcs ${P}_{T}T$ and ${P}_{B}$ H are always 90°. Thus, the arc ${P}_{B}$ T can be found by subtracting δ from 90°.

The elevation angle *δ* is derived from the spherical triangle $\Delta {P}_{B}{P}_{T}T$. By applying the law of cosines for sides to the spherical triangle, we find that

14 $$\begin{array}{c}\mathrm{cos}\left(90^\circ -\delta \right)=\mathrm{cos}{i}_{R }\mathrm{cos}90^\circ +\mathrm{sin}{i}_{R }\mathrm{sin}90^\circ \mathrm{cos}\left(90^\circ -{\theta }_{T}\right) \\ \mathrm{sin}\delta =\mathrm{sin}{i}_{R }\mathrm{sin}{\theta }_{T}\end{array} $$

Thus, angle *δ* is obtained by

15 $$\delta ={\mathrm{sin}}^{-1}\left[\mathrm{sin}{i}_{R }\mathrm{sin}{\theta }_{T}\right]$$

The azimuth angle *α* is found by applying the law of cosines for sides twice to the spherical triangle $\Delta \mathrm{T}H{I}_{P}$, resulting in the following equations:

16 $$\mathrm{cos}{\theta }_{T}=\mathrm{cos}\delta \mathrm{cos}\left({\theta }_{B}+\alpha \right)+\mathrm{sin}\delta \mathrm{sin}\left({\theta }_{B}+\alpha \right) \mathrm{cos}90^\circ $$

and

17 $$\mathrm{cos}\delta =\mathrm{cos}{\theta }_{T }\mathrm{cos}\left({\theta }_{B}+\alpha \right)+\mathrm{sin}{\theta }_{T}\mathrm{ sin}\left({\theta }_{B}+\alpha \right) \mathrm{cos}{i}_{R}$$

Substituting $\mathrm{cos}\delta$ from Eqs. (16) into (17), we obtain

18 $$\mathrm{tan}\left({\theta }_{B}+\alpha \right) = \frac{\mathrm{sin}{\theta }_{T }\mathrm{cos}{i}_{R}}{\mathrm{cos}{\theta }_{T}}$$

Thus, angle *α* applying the full-sky trigonometry solutions is derived by

19 $$\alpha =-{\theta }_{B}+\mathrm{atan}2 \left[ \mathrm{sin}{\theta }_{T}\mathrm{ cos}{i}_{R}, \mathrm{cos}{\theta }_{T}\right]$$

Using the definition of the argument of latitudes in Eq. (5), angles *α* and *δ* are expressed as

20 $$ \begin{array}{*{20}l} {\alpha = \left( {\phi_{B} - \omega_{B} - \nu_{B} } \right) + {\text{atan}}2{ }\left[ {{\text{cos}}i_{R } {\text{sin}} \left( {\omega_{T} + \nu_{T} - \phi_{T} } \right), {\text{cos }}\left( {\omega_{T} + \nu_{T} - \phi_{T} } \right) } \right], } \hfill & {0^\circ \le \alpha < 360^\circ } \hfill \\ { \delta = {\text{sin}}^{ - 1} \left[ {{\text{sin}}i_{R} {\text{sin}}\left( {\omega_{T} + \nu_{T} - \phi_{T} } \right)} \right], } \hfill & { - 90^\circ \le \delta \le 90^\circ } \hfill \\ \end{array} $$

### Note 2

The quadrant ambiguity problem is avoided by using the ATAN2 built-in function in computer programming languages for ${\phi }_{B},{\phi }_{T},\alpha$, in order to cover the full range of 0° to 360°.

For a simple analysis of satellite relative motion, angles *α* and *δ* can be directly used to determine the angular position of the target satellite with respect to the base satellite. In Eq. (20), *ν* is the only time-dependent variable, and the derivatives of *α* and *δ* are obtained using:

21 $$\begin{array}{c}\dot{\alpha }={\mathrm{cos}}{i}_{R}\left(1+{\mathrm{tan}}^{2}\delta \right){\dot{v}}_{T}-{\dot{v}}_{B}\\ \dot{\delta }={\mathrm{sin}}{i}_{R}{\mathrm{cos}}\left(\alpha +{\nu }_{B}+{\omega }_{B}-{\phi }_{B}\right){\dot{v}}_{T}\end{array}$$

The angle *δ* in Eq. (20) is a composite function and the derivative of *δ* for Eq. (21) is computed using the chain rule. The relative motion of the target satellite can be described using the previously calculated *α* and *δ* in the rectangular coordinates. The orbit radius of the base satellite is ${r}_{B}$ and the target satellite orbit radius is ${r}_{T}$. We introduce the base satellite rotating frame, ${F}_{R}$, to describe the relative motion of the target satellite with respect to the base satellite. The center of the Earth is set as the origin, and the orientation of ${F}_{R}$ is given by the unit vectors ${\widehat{e}}_{1},{\widehat{e}}_{2},{ \mathrm{and} \widehat{e}}_{3}$. The direction of the unit vector ${\widehat{e}}_{1}$ is set to the orbit radius of the base satellite, while ${\widehat{e}}_{3}$ is perpendicular to the orbit plane of the base satellite. The unit vector ${\widehat{e}}_{2}$ is then computed following the right-hand rule. ${F}_{R}$ is mathematically described by the unit vectors as follows:

22 $$\begin{array}{c}{\widehat{e}}_{1} = \frac{{r}_{B}}{\left|{r}_{B}\right|}\\ { \widehat{e}}_{3} = \frac{{r}_{B}^{\times }{\dot{r}}_{B}}{\left|{r}_{B}^{\times }{\dot{r}}_{B}\right|}\\ {\widehat{e}}_{2} = {e}_{3}^{\times }{\widehat{e}}_{1}\end{array}$$

Superscript × denotes a skew-symmetric $3 \times 3$ matrix associated with a $3\times 1$ column matrix. If $x$ is a $3\times 1$ matrix, $x$= ${\left[{x}_{1} {x}_{2} {x}_{3}\right]}^{T}$, then:

23 $${x}^{\times } = \left[\begin{array}{ccc}0& -{x}_{3}& {x}_{2}\\ {x}_{3}& 0& -{x}_{1}\\ -{x}_{2}& {x}_{1}& 0\end{array}\right]$$

The position vectors of the base and target satellites can be written as the vector components in ${F}_{R}$:

24 $$\begin{array}{c}{\overrightarrow{r}}_{B} = {\left({r}_{B} 0 0\right)}^{T} \\ {\overrightarrow{r}}_{T}= {\left({r}_{T}\mathrm{ cos}\delta \mathrm{cos}\alpha {r}_{T}\mathrm{ cos}\delta \mathrm{cos}\alpha {r}_{T}\mathrm{ sin}\delta \right)}^{T}\end{array}$$

The relative position vector $\overrightarrow{r}$ of the target satellite in the base satellite centered frame, ${F}_{C}$ (with the base satellite as the origin), is derived by vector subtraction of the position vectors from Eq. (24):

25 $$\stackrel{\rightharpoonup }{r} = \left(\begin{array}{c}x\\ y\\ z\end{array}\right)= \left(\begin{array}{c}{r}_{T} cos\delta cos\alpha - {r}_{B}\\ {r}_{T }cos\delta sin\alpha \\ {r}_{T }sin\delta \end{array}\right)$$

The relative velocity vector $\overrightarrow{v}$ is obtained by the time derivatives of Eq. (25):

26 $$\stackrel{\rightharpoonup }{v} = \left(\begin{array}{c}\dot{x}\\ \dot{y}\\ \dot{z}\end{array}\right) = \left(\begin{array}{c}{\dot{r}}_{T} cos\delta cos\alpha -{r}_{T }\dot{\delta }sin\delta cos\alpha -{r}_{T }\dot{\alpha } cos\delta sin\alpha -{\dot{r}}_{B}\\ {\dot{r}}_{T} cos\delta sin\alpha -{r}_{T }\dot{\delta } sin\delta sin\alpha {+r}_{T}\dot{ \alpha } cos\delta cos\alpha \\ {\dot{r}}_{T }sin\delta +{r}_{T} \dot{\delta } cos\delta \end{array}\right)$$

where the derivatives of *r* and *ν* of the satellites are (Schaub and Junkins^19^)

27 $${\dot{r}}_{j}= \sqrt{\frac{\mu }{{a}_{j}\left(1-{e}_{j}^{2}\right)}{e}_{j}\mathrm{sin}{\nu }_{j}}, {\dot{v}}_{j}=\frac{\sqrt{\mu {a}_{j}\left(1-{e}_{j}^{2}\right)}}{{r}_{j}^{2}}, j=B, T$$

The relative equations of motion [Eqs. (25) and (26)] are an exact analytic solution for satellite relative motions plugging in the variables of Eqs. (20) and (21). The only assumption is that no perturbations are acting on the satellites.

The resulting solutions, we collectively call herein as geometrical relative orbit modeling (GROM), are obtained by the direct geometrical method using full-sky spherical geometry. GROM has the following explicit advantages: first, the angular values ($\alpha ,\delta ,\dot{\alpha },\dot{\delta }$) are useful for the angle-only measurement analysis or tracking problems between satellites without any coordinate transformation; and second, the solutions provide a comparatively simpler form for the dynamic model of relative orbits. For comparison, this study introduced the USA (see Online Appendix for this approach)^10^, which nonlinearly maps orbital elements to the relative position and velocity in the same manner as GROM. The two solutions consisted of trigonometric functions as orbital element terms. Table 1 presents a comparison of the formula complexity of the solutions.

**Table 1:** Formula complexity of the two solutions.

GROM	USA
The solution is comparatively simple (49 trigonometry functions)	This requires an efficient method for simple-form expressions (82 trigonometry functions)

The GROM is expressed in compact form with the ATAN 2 inverse trig function, and the USA represents analytic expressions in terms of differential orbital elements, along with the direction cosine matrix of the chief and deputy satellites.

## Model evaluations

We first evaluate the modeling accuracy to determine the validity of the GROM solution. To evaluate the modeling accuracy, three relative motion theories are introduced: Hill’s equation^19^, the classical two-body problem (CTBP)^20^, and the USA. The USA has been evaluated to have the lowest index in terms of accuracy of satellite relative motion equivalent to the Yan–Alfriend nonlinear method^8,10^.

### Model accuracy

This section evaluates the relative errors of GROM using the modeling error index which is an effective tool for evaluating the accuracy of relative motion theories^8^. In Eq. (28), ${\stackrel{-}{\mathrm{x}}}_{j}$ and ${\mathrm{x}}_{j}$ are the relative position and velocity vectors of the reference and proposed model, respectively:

28 $${\overline{y}}_{j} = W{\overline{x}}_{j}, \quad {y}_{j} = W{x}_{j}$$

where *j* represents each sample point of a relative orbit. The weighting matrix $W$ uses the Earth-value units as shown in Eq. (29):

29 $$W = diag\left(\frac{1}{{R}_{e}},\frac{1}{{R}_{e}},\frac{1}{{R}_{e}},\frac{1}{{R}_{e}n},\frac{1}{{R}_{e}n},\frac{1}{{R}_{e}n}\right)$$

where ${R}_{e}$ is the Earth’s radius of 6378.14 km and *n* is the mean motion of satellite. The modeling error index is written as follows:

30 $$\begin{array}{c}{\lambda }_{j} = \frac{{\overline{y}}_{j}^{T}{\overline{y}}_{j}}{{y}_{j}^{T}{y}_{j}} - 1\\ \lambda = \underset{j=1..m}{max}\left|{\lambda }_{j}\right|\end{array}$$

The modeling error index evaluates the relative errors of GROM in comparison to Hill’s equation and USA, relative to the reference orbit model of CTBP. The analytic solutions of USA and CTBP describe the kinematically exact relative motion of satellites, which brings about negligible quantities of relative errors upon simulation. We use *k*-digit rounding arithmetic when finding the solutions, ${\mathrm{y}}_{j}$ and ${\stackrel{-}{\mathrm{y}}}_{j}$, to ignore computational uncertainties such as round-off errors. The *k*-digit rounding arithmetic is obtained by terminating the value of the solution at *k* decimal digits.

For numerical simulations, the orbit elements of the base satellite in Table 2 are chosen, and the orbit element differences, $\Delta \mathrm{oe}$, of the target satellite are used as the following values:

**Table 2:** Parameter of the orbit elements.

Orbit elements	Value	Units
a	7000	km
e	0.001	–
i	30.0	deg
Ω	120.0	deg
ω	0.0	deg
${M}_{0}$	0.0	deg

31 $$\begin{array}{c}\Delta oe = \left[\Delta a \Delta e 0.1 0.2 0.01 0.0\right] \\ \Delta a = \left[0.0 0.001 0.005 0.01 0.1 0.5 5\right] \\ \Delta e = \left[0.0 0.00001 0.00005 0.0001 0.0005 0.05 0.1\right]\end{array}$$

**Figure 6:**
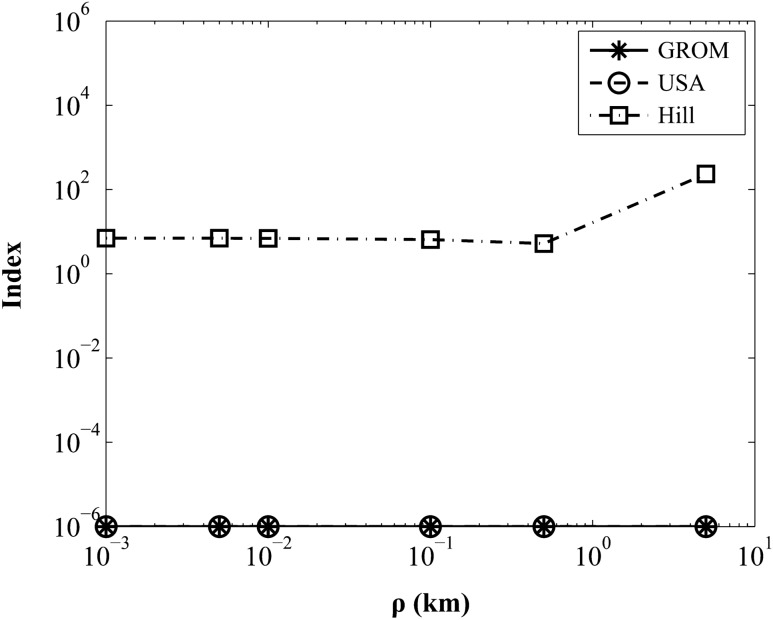
Index Comparison for various relative distances.

**Figure 7:**
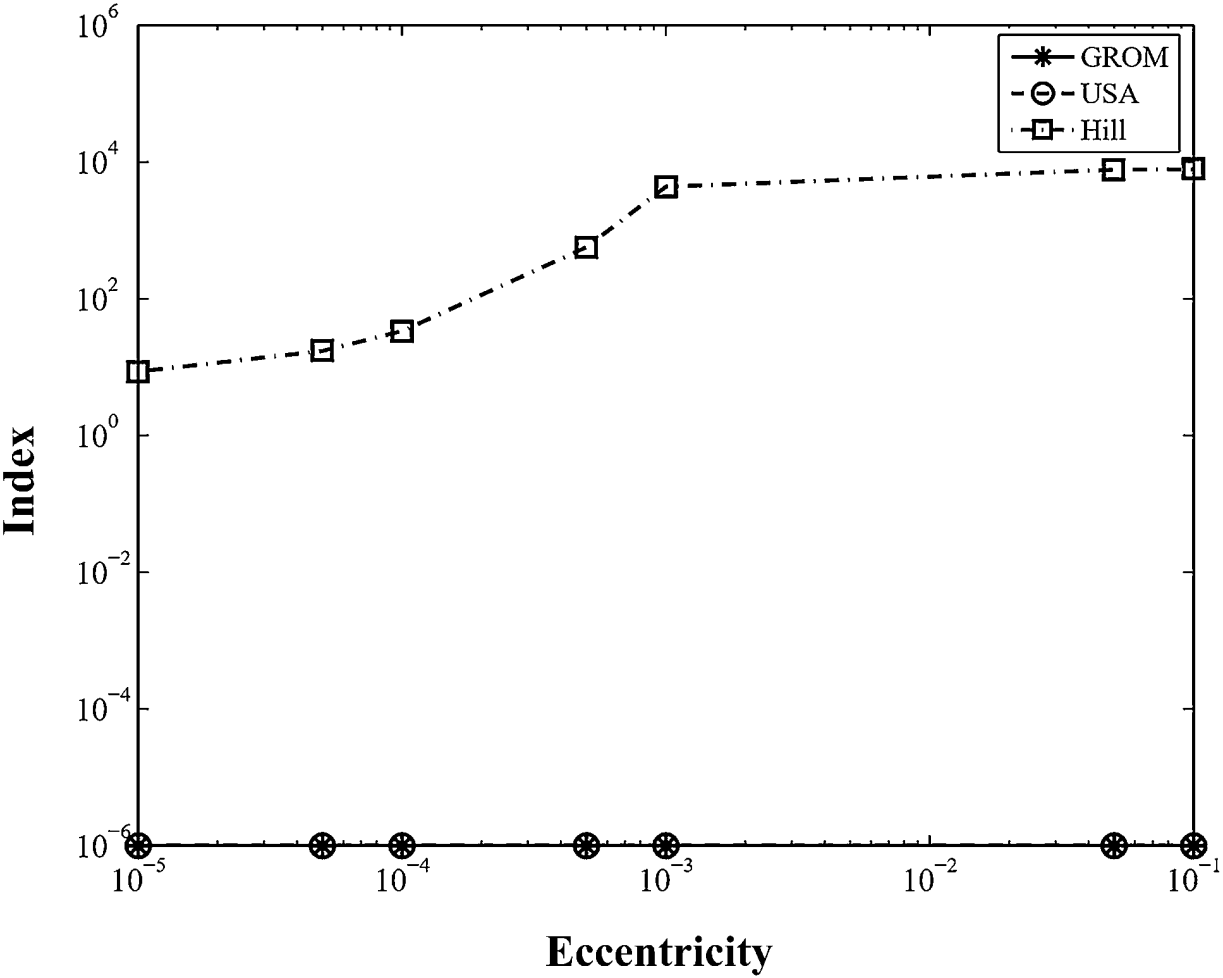
Index Comparison for various eccentricities.

Figures 6 and 7 show the modeling error index using the six-digit rounding arithmetic solution with various relative distances and eccentricities. As shown in the figures, the index of GROM is exactly the same as that of USA, representing index values of 10^−6^ with respect to the reference orbit model. A modeling error index of the order of 10^−3^ is sufficiently small, providing a reasonable confidence regarding the modeling^21^.

Therefore, the GROM solution provides an accurate representation for all relative orbit sizes and eccentricities. In the case of the Hill’s equation, the solution shows modeling indexes of nearly 10^1^ at small orbit element differences, which means sufficient accuracy for small relative orbit sizes and eccentricities. As expected, however, the index values gradually grow with increasing orbit size and eccentricity.

### Model efficiency

This section compares CTBP of a vector solution, and USA of a nonlinear mapping solution similar to GROM, to evaluate the computational cost of GROM. In tests, we used MATLAB (R2013b) running on an 8th Intel Core i5 8265U (3.4 GHz) with 8 GB memory. The CPU time comparison of each solution is clearly implementation-dependent on the researcher. To fairly evaluate the comparison of solutions, each solution was coded efficiently, and a simple least squares linear regression and method was applied for a more robust comparison. Generally, the relationship between iterations, $\stackrel{\sim }{N,}$ and CPU time, $\stackrel{\sim }{T,}$ is roughly linear. However, it is not exact linear. Along with the parameters in Table 2, five iterations, were performed with a time step 0.1 s and a time span(sec) below:

32 $$\tilde{N } = \left[10 10000 30000 50000 100000\right]$$

Iteration and CPU time are evaluated by the following linear model:

33 $${\tilde{T }}_{k} = m {\tilde{N }}_{k}, k=1,\dots ,n$$

The computational cost of each solution is compared by the slope *m* value and the least squares method is used to calculate the slope of the best fit line. Table 3 shows the coefficient *m*^∗^, normalized with respect to the value of GROM. GROM is nearly 7% and 25% more efficient than USA and CTBP, respectively.

**Table 3:** Comparison of analytic solution efficiency.

Method	GROM	USA	CTBP
Normalized coefficient (m∗)	1.0000	1.0711	1.2524

A numerical example was used to study the effect of the satellite relative motion under the influence of ${J}_{2}$ perturbations, through which we demonstrated the modeling efficiency of GROM. Using the time-explicit orbital elements in the analytic solutions provides a simple method of investigating the difference between unperturbed and ${J}_{2}$-perturbed models for the satellite relative motion. The first-order ${J}_{2}$ perturbation causes secular changes in the ascending node, Ω, argument of perigee, *ω*, and mean anomaly, *M*. The time-explicit representations of the first-order ${J}_{2}$ perturbation are presented as follows^10^:

34 $$\begin{array}{c}a = {a}_{0} \\ e = {e}_{0} \\ i = {i}_{0} \\ \Omega = {\Omega }_{0 }- \frac{3n{R}_{e}^{2}{J}_{2}\mathrm{cos}i}{2{p}^{2}}t \\ \omega = {\omega }_{0 }- \frac{3n{R}_{e}^{2}{J}_{2}}{4{p}^{2}}(4-5{\mathrm{sin}}^{2}i)t \\ M = {M}_{0 }+nt+ \frac{3n{R}_{e}^{2}{J}_{2}\sqrt{1-{e}^{2}}}{4{p}^{2}}(3{\mathrm{sin}}^{2}i-2)t\end{array}$$

We use the following values in the time-explicit orbit elements: *p* = *a*(1 − *e*^2^), ${J}_{2}$ = 0.00108263.

The numerical simulation coded to iterate the solutions at each time step runs over a period of 20 days using the orbit elements for the base satellite shown in Table 2. The orbit element differences are chosen as the following values:

35 $$\Delta oe = \left[0.0 0.0001 0.01 0.02 0.01 0.02\right]$$

Table 4 shows that the maximum differences between the relative position and velocity of the solutions are 3*.*8729 km and 0*.*0041 km/s over 20 days, respectively. However, each of the analytic solution results in different CPU times; thus, the GROM solution is approximately ${1}^{^{\prime}}{40}^{{^{\prime}}{^{\prime}}}$ and ${5}^{^{\prime}}{57}^{{^{\prime}}{^{\prime}}}$ faster than the USA and CTBP solutions, respectively.

**Table 4:** Differences between the unperturbed and *J*_2_ perturbed models (time step: 0.1 s).

Methods	CPU time, (min)	Maximum position difference, (km)	Maximum velocity difference, (km/s)
GROM	23.60	3.8729	0.0041
USA	25.27	3.8729	0.0041
CTBP	29.55	3.8729	0.0041

## Conclusions

Herein, we developed an analytic solution for the unperturbed satellite relative motion using a direct geometrical approach on a sphere. The derivation of this geometrical approach is straightforward, and the resulting solutions provide a complete analytic form for relative motion dynamics with the full-sky solutions covering the range of 0°–360°. The accuracy of the proposed GROM solution equals that of the exact relative motion theories of CTBP and USA. In particular, in terms of the modeling efficiency, GROM is approximately 7% and 25% more efficient than USA and CTBP, respectively. Consequently, using full-sky spherical geometry, the proposed GROM approach facilitates simpler equations of motion and higher computational speed compared with the other methods.

## Supplementary Information

Supplementary Information.
