# Supplementary material for: An analytic solution of full-sky spherical geometry for satellite relative motions
Source: Sci Rep. 2021 Apr 27;11:9075. doi: 10.1038/s41598-021-88483-2 (PMC8079416; doi:10.1038/s41598-021-88483-2)
Supplement: Supplementary file 1 — Supplementary Information. [file 41598_2021_88483_MOESM1_ESM.docx]

# Appendix

## Unit Sphere Approach

The relative position on the unit sphere is expressed as follows:

$\left( \begin{aligned} \Delta x \\ \Delta y \\ \Delta z \end{aligned} \right) = \left[ R_{1}R_{2}^{T} - I \right]\left( \begin{aligned} 1 \\ 0 \\ 0 \end{aligned} \right)$ (A1)

where $\Delta x, \Delta y, \mathrm{and} \Delta z$ are the radial, in-track, and cross-track relative positions of the target satellite on the unit sphere, respectively. The direction cosine matrix,$R_{j}$, of the base and target satellites is provided as follows:

$R_{j} = \left[ \begin{matrix} \cos u_{j}\cos\Omega_{j}-\cos i_{j}\sin u_{j}\sin\Omega_{j} & \cos i_{j}\cos\Omega_{j}\sin u_{j}-\cos u_{j}\sin\Omega_{j} & \sin i_{j}\sin u_{j} \\ -\sin u_{j}\cos\Omega_{j}-\cos i_{j}\cos u_{j}\sin\Omega_{j} & \cos i_{j}\cos u_{j}\cos\Omega_{j}-\sin u_{j}\sin\Omega_{j} & \sin i_{j}\cos u_{j} \\ \sin i_{j}\sin\Omega_{j} & -\sin i_{j}\cos\Omega_{j} & \cos i_{j} \end{matrix} \right]$ (A2)

The relative positions of the unit sphere approach can be expanded as follows:

$\begin{aligned} \Delta x=-1+\cos^{2}\left( 0.5i_{1} \right)\cos^{2}\left( 0.5i_{2} \right)\cos\left( u_{2}-u_{1}+\Omega_{2}-\Omega_{1} \right) \\ +\sin^{2}\left( 0.5i_{1} \right)\sin^{2}\left( 0.5i_{2} \right)\cos\left( u_{2}-u_{1}-\Omega_{2}+\Omega_{1} \right) \\ +\sin^{2}\left( 0.5i_{1} \right)\cos^{2}\left( 0.5i_{2} \right)\cos\left( u_{2}+u_{1}+\Omega_{2}-\Omega_{1} \right) \\ +\cos^{2}\left( 0.5i_{1} \right)\sin^{2}\left( 0.5i_{2} \right)\cos\left( u_{2}+u_{1}-\Omega_{2}+\Omega_{1} \right) \\ +0.5\sin i_{1}\sin i_{2}\left[ \cos\left( u_{2}-u_{1} \right)-\cos\left( u_{2}+u_{1} \right) \right] \\ \end{aligned}$ $\begin{aligned} \Delta y=\cos^{2}\left( 0.5i_{1} \right)\cos^{2}\left( 0.5i_{2} \right)\sin\left( u_{2}-u_{1}+\Omega_{2}-\Omega_{1} \right) \\ +\sin^{2}\left( 0.5i_{1} \right)\sin^{2}\left( 0.5i_{2} \right)\sin\left( u_{2}-u_{1}-\Omega_{2}+\Omega_{1} \right) \\ -\sin^{2}\left( 0.5i_{1} \right)\cos^{2}\left( 0.5i_{2} \right)\sin\left( u_{2}+u_{1}+\Omega_{2}-\Omega_{1} \right) \\ -\cos^{2}\left( 0.5i_{1} \right)\sin^{2}\left( 0.5i_{2} \right)\sin\left( u_{2}+u_{1}-\Omega_{2}+\Omega_{1} \right) \\ +0.5\sin i_{1}\sin i_{2}\left[ \sin\left( u_{2}-u_{1} \right)-\sin\left( u_{2}+u_{1} \right) \right] \\ \end{aligned}$

$$\Delta z=-\sin i_{1}\sin\left( \Omega_{2}-\Omega_{1} \right)\cos u_{2}-\left[ \sin i_{1}\cos i_{2}\cos\left( \Omega_{2}-\Omega_{1} \right)-\cos i_{1}\sin i_{2} \right]\sin u_{2}$$

(A3)

The relative velocities of the unit sphere approach are given as follows:

$$\begin{aligned} \Delta\dot{x}=-\cos^{2}\left( 0.5i_{1} \right)\cos^{2}\left( 0.5i_{2} \right)\sin\left( u_{2}-u_{1}+\Omega_{2}-\Omega_{1} \right)\left( \dot{v}_{2}-\dot{v}_{1} \right) \\ -\sin^{2}\left( 0.5i_{1} \right)\sin^{2}\left( 0.5i_{2} \right)\sin\left( u_{2}-u_{1}-\Omega_{2}+\Omega_{1} \right)\left( \dot{v}_{2}-\dot{v}_{1} \right) \\ -\sin^{2}\left( 0.5i_{1} \right)\cos^{2}\left( 0.5i_{2} \right)\sin\left( u_{2}+u_{1}+\Omega_{2}-\Omega_{1} \right)\left( \dot{v}_{2}+\dot{v}_{1} \right) \\ -\cos^{2}\left( 0.5i_{1} \right)\sin^{2}\left( 0.5i_{2} \right)\sin\left( u_{2}+u_{1}-\Omega_{2}+\Omega_{1} \right)\left( \dot{v}_{2}+\dot{v}_{1} \right) \\ -0.5\sin i_{1}\sin i_{2}\left[ \sin\left( u_{2}-u_{1} \right)\left( \dot{v}_{2}-\dot{v}_{1} \right)-\sin\left( u_{2}+u_{1} \right)\left( \dot{v}_{2}+\dot{v}_{1} \right) \right] \end{aligned}$$

$$\begin{aligned} \Delta\dot{y}=\cos^{2}\left( 0.5i_{1} \right)\cos^{2}\left( 0.5i_{2} \right)\cos\left( u_{2}-u_{1}+\Omega_{2}-\Omega_{1} \right)\left( \dot{v}_{2}-\dot{v}_{1} \right) \\ +\sin^{2}\left( 0.5i_{1} \right)\sin^{2}\left( 0.5i_{2} \right)\cos\left( u_{2}-u_{1}-\Omega_{2}+\Omega_{1} \right)\left( \dot{v}_{2}-\dot{v}_{1} \right) \\ -\sin^{2}\left( 0.5i_{1} \right)\cos^{2}\left( 0.5i_{2} \right)\cos\left( u_{2}+u_{1}+\Omega_{2}-\Omega_{1} \right)\left( \dot{v}_{2}+\dot{v}_{1} \right) \\ -\cos^{2}\left( 0.5i_{1} \right)\sin^{2}\left( 0.5i_{2} \right)\cos\left( u_{2}+u_{1}-\Omega_{2}+\Omega_{1} \right)\left( \dot{v}_{2}+\dot{v}_{1} \right) \\ +0.5\sin i_{1}\sin i_{2}\left[ \sin\left( u_{2}-u_{1} \right)\left( \dot{v}_{2}-\dot{v}_{1} \right)+\cos\left( u_{2}+u_{1} \right)\left( \dot{v}_{2}+\dot{v}_{1} \right) \right] \end{aligned}$$

$$\Delta\dot{z}=\sin i_{1}\sin\left( \Omega_{2}-\Omega_{1} \right)\sin u_{2}\dot{v}_{2}-\left[ \sin i_{1}\cos i_{2}\cos\left( \Omega_{2}-\Omega_{1} \right)-\cos i_{1}\sin i_{2} \right]\cos u_{2}\dot{v}_{2}$$

(A4)

The actual relative motion between the two satellites is written as follows:

$\begin{aligned} x=r_{2}\left( 1+\Delta x \right)-r_{1} \\ y= r_{2}\Delta y \\ z= r_{2}\Delta z \end{aligned}$ (A5)

The relative velocity vectors of the target satellite are expressed as follows:

$\begin{aligned} \dot{x}=\dot{r_{2}}\left( 1+\Delta x \right)+r_{2}\Delta\dot{x}-\dot{r_{1}} \\ \dot{y} = \dot{r_{2}}\Delta y+r_{2}\Delta\dot{y} \\ \dot{z} = \dot{r_{2}}\Delta z+r_{2}\Delta\dot{z} \end{aligned}$ (A6)
